# Phylogeography and allopatric divergence of cypress species (*Cupressus *L.) in the Qinghai-Tibetan Plateau and adjacent regions

**DOI:** 10.1186/1471-2148-10-194

**Published:** 2010-06-22

**Authors:** Tingting Xu, Richard J Abbott, Richard I Milne, Kangshan Mao, Fang K Du, Guili Wu, Zhaxi Ciren, Georg Miehe, Jianquan Liu

**Affiliations:** 1 Division of Molecular Ecology, Key Laboratory of Arid and Grassland Ecology, School of Life Science, Lanzhou University, Lanzhou, 730000, China; 2 School of Biology, Mitchell Building, University of St Andrews, St Andrews, Fife KY16 9TH, UK; 3 Institute of Molecular Plant Sciences, The University of Edinburgh, Daniel Rutherford Building, King's Buildings, Mayfield Road, Edinburgh EH9 3JH, UK; 4 Department of Life Science, Tibet University, Lasha, Tibet, China; 5 Faculty of Geography, University of Marburg, Deutschhaustr.10 35032 Marburg, Germany

## Abstract

**Background:**

Although allopatric speciation is viewed as the most common way in which species originate, allopatric divergence among a group of closely related species has rarely been examined at the population level through phylogeographic analysis. Here we report such a case study on eight putative cypress (*Cupressus*) species, which each have a mainly allopatric distribution in the Qinghai-Tibetan Plateau (QTP) and adjacent regions. The analysis involved sequencing three plastid DNA fragments (*trn*D-*trn*T, *trn*S-*trn*G and *trn*L-*trn*F) in 371 individuals sampled from populations at 66 localities.

**Results:**

Both phylogenetic and network analyses showed that most DNA haplotypes recovered or haplotype-clustered lineages resolved were largely species-specific. Across all species, significant phylogeographic structure (*N*_ST _> *G*_ST_, *P *< 0.05) implied a high correlation between haplotypes/lineages and geographic distribution. Two species, *C. duclouxiana *and *C. chengiana*, which are distributed in the eastern QTP region, contained more haplotypes and higher diversity than five species with restricted distributions in the western highlands of the QTP. The remaining species, *C. funebris*, is widely cultivated and contained very little cpDNA diversity.

**Conclusions:**

It is concluded that the formation of high mountain barriers separating deep valleys in the QTP and adjacent regions caused by various uplifts of the plateau since the early Miocene most likely promoted allopatric divergence in *Cupressus *by restricting gene flow and fixing local, species-specific haplotypes in geographically isolated populations. The low levels of intraspecific diversity present in most species might stem from population bottlenecks brought about by recurrent periods of unfavorable climate and more recently by the negative impacts of human activities on species' distributions. Our findings shed new light on the importance of geographical isolation caused by the uplift of the QTP on the development of high plant species diversity in the QTP biodiversity hotspot.

## Background

Geographic isolation between populations within species is a major precursor of allopatric speciation [1,2]. In the absence of gene flow, different alleles become fixed in different populations through the action of selection and/or genetic drift potentially leading to the origin of new species [3]. It is expected, therefore, that in regions where geographic isolation is common, for example, in areas where high mountains separate populations occurring in deep valleys, allopatric divergence will be promoted and accelerated due to the formation of locally adapted gene pools and possibly the origin of intrinsic postzygotic isolation according to the Bateson-Dobzhansky-Muller model [3-5]. A phylogeographic analysis of closely related species that occur in such areas can be instructive on the importance of geographic isolation and allopatric divergence in their origin and subsequent evolution [6].

**Figure 1:**
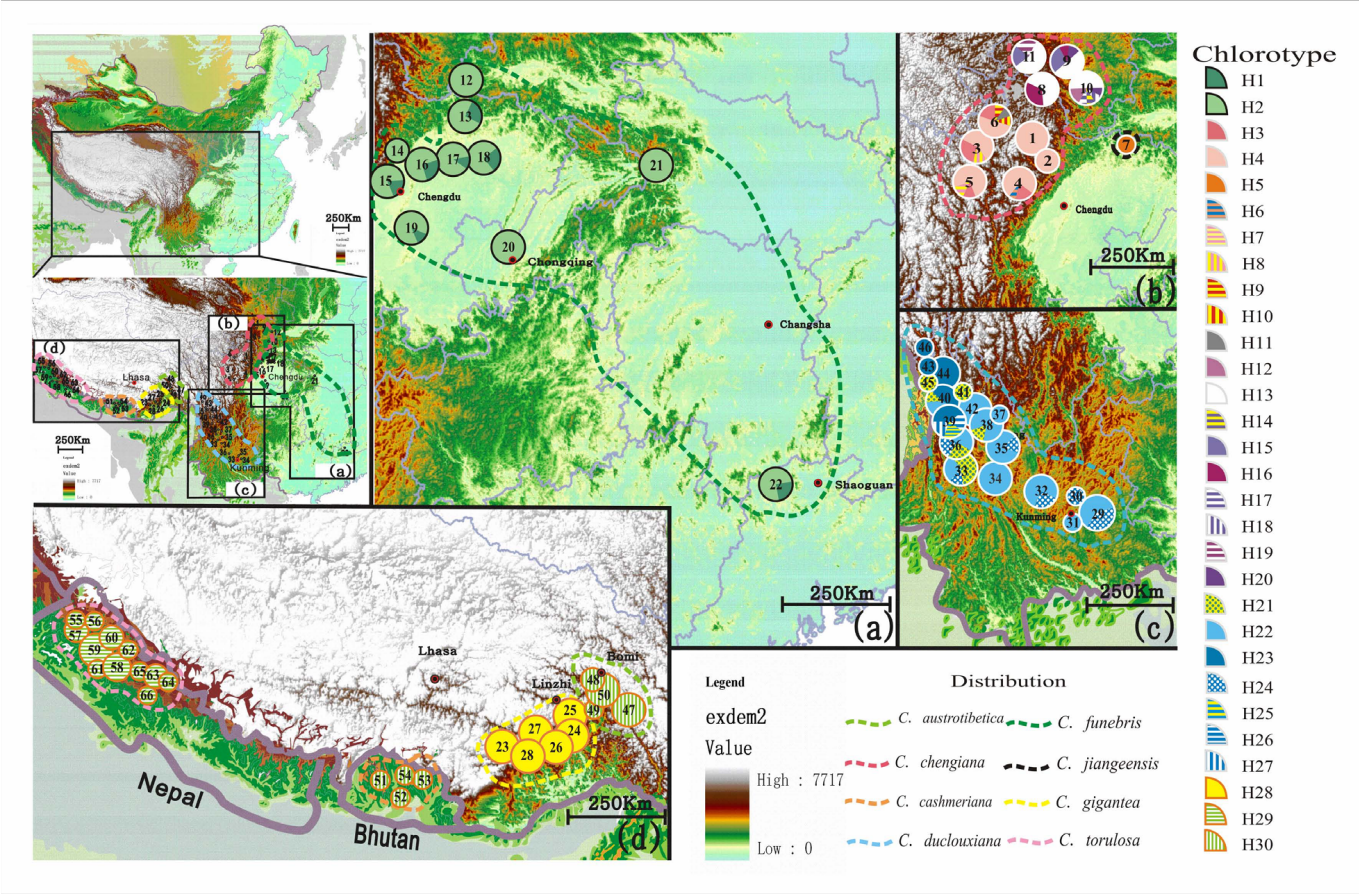
**The distribution of eight cypress species and frequencies of plastid DNA haplotypes in each population surveyed**. Upper left panel, a, b, c and d: Geographic ranges of eight cypress species and locations of populations sampled. The upper right panels a, b, c and lower panel d: plastid DNA haplotypes identified and the proportion of each present within populations of each species examined (circle size is proportional to the number of individuals surveyed per population). Further details of size and haplotype diversity of populations are provided in Additional File 1.

The Qinghai-Tibetan Plateau (QTP) and adjacent regions comprise an excellent mountainous system for studying plant diversification and evolution. Levels of plant diversity and endemism in these regions are very high and some core areas are regarded as biodiversity hotspots (e.g. [7]). A few studies in this region have begun to elucidate the process of species diversification and/or trace shifts in the ranges of single species during the Quaternary climatic oscillations (e.g. [8,9]). However, no previous studies have attempted to examine genetic differentiation in this region among species of a closely related group, and in so doing shed light on their respective evolutionary histories. Here we report such a case study of a group of cypress species (*Cupressus *L.) that occurs on the QTP and adjacent areas of China and the Himalayas. Cypresses are trees or shrubs native to scattered localities in temperate regions in the northern hemisphere. Because of their attractive morphology they are important in horticulture and are widely cultivated in many parts of the world [10]. In several mountainous regions of the QTP, cypresses form natural forests [11]. From the results of a recent molecular phylogenetic analysis of cypresses, Little [12] concluded that True Cypresses only encompass species in the Old World, and that those in the New World should be transferred to *Callitropsis*. As a result, the genus *Cupressus *has been re-circumscribed to comprise 12 Old World species, distributed respectively in the Mediterranean region and Africa (*C. sempervirens*, *C. dupreziana *and *C. atlantica*) and Asia (*C. torulosa*, *C. gigantea*, *C. cashmeriana *and *C. austrotibetica *in the high altitude region of the QTP and west Himalayas, and *C. duclouxiana*, *C. chengiana*, *C. funebris*, *C. jiangeensis *and *C. tonkinensis *in the low altitude region of the eastern QTP, central China and Vietnam [12]). Morphological distinction between some of these species is marginal causing taxonomic delimitation to be disputed, especially between Asian species [10,12-19]. We followed Little [12] and treated all taxonomic units recognized by him as separate species. Most of these Asian species occur allopatrically in the QTP and adjacent regions, and only a few are parapatric in parts of their range (Fig. 1) (Additional File 1) [10,15].

We used plastid (mainly chloroplast) DNA sequence data to examine interspecific divergence and to assess intraspecific diversity of these species. Plastid DNA is paternally inherited and dispersed mainly by pollen in all species of the Cupressaceae investigated [20-23]. Recent studies suggest that paternally inherited plastid DNA sequences in conifers may be species-specific and therefore effective in tracing lineage sorting between species [24-27]. Plastid DNA sequence variation has been used previously in phylogenetic studies of cypresses, but only single sample from most species were examined [12]. In addition, surveys of RAPD variation [28] and nuclear ITS variation [29] have been conducted within and among cypress species including those examined in the current study. However, the results of these studies were inconsistent with regard to interspecific relationships [12]. In this study, we aimed to address the following questions based on surveys of sequence variation for three plastid DNA fragments within and between populations at the species level: (1) Do plastid DNA haplotypes or clusters of such haplotypes correspond with morphological differentiation and the allopatric distributions of the *Cupressus *species examined? (2) Does intraspecific DNA diversity correlate with the natural population size of a species and, in turn, provide guidance for conservation management?

## Results

### Haplotype distribution and relationships

**Figure 2:**
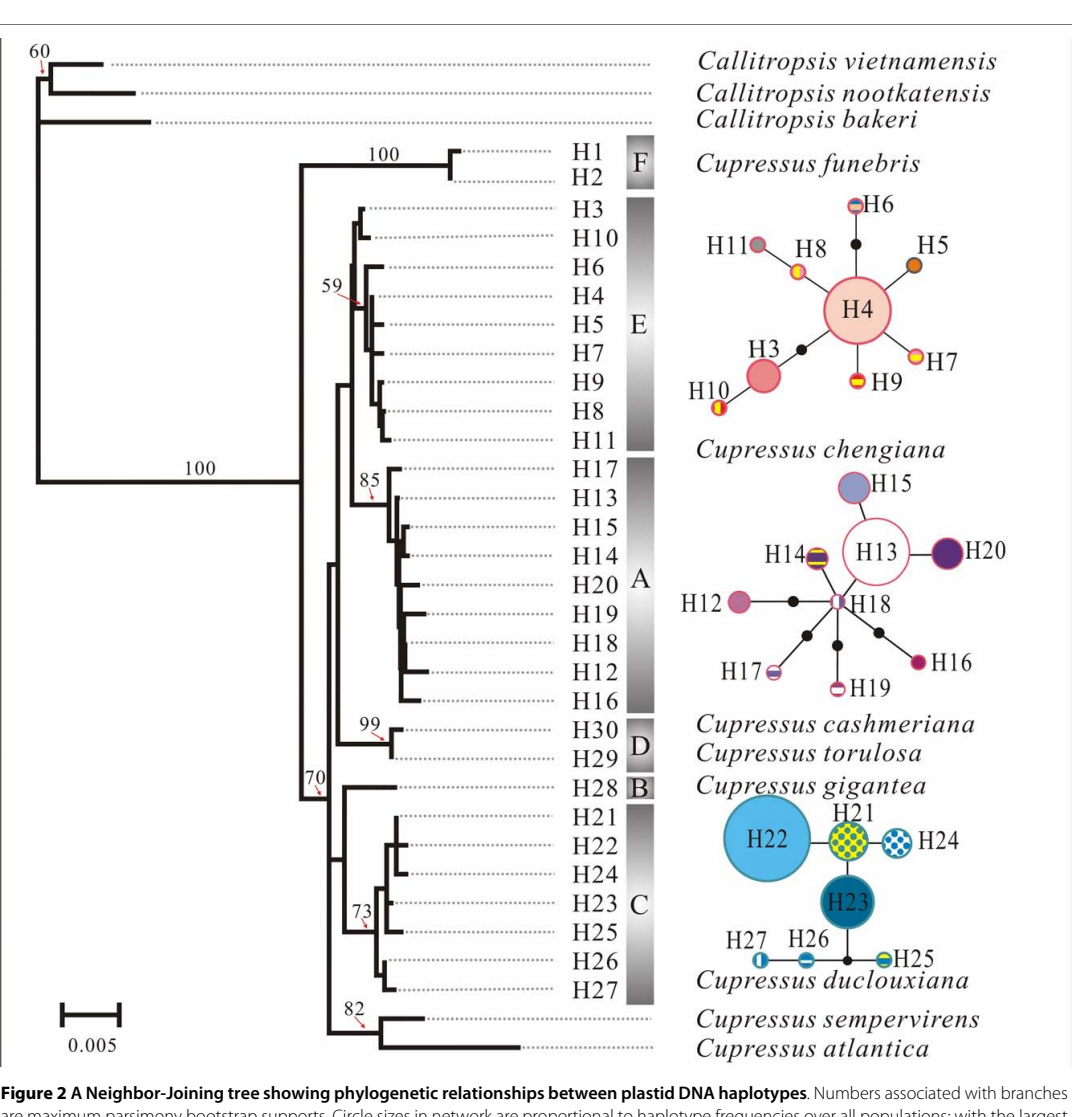
**A Neighbor-Joining tree showing phylogenetic relationships between plastid DNA haplotypes**. Numbers associated with branches are maximum parsimony bootstrap supports. Circle sizes in network are proportional to haplotype frequencies over all populations; with the largest circle representing the most abundant haplotype and the colours of circles indicating different haplotypes as shown in Fig. 1.

In total, 30 haplotypes were identified (Additional Files 1, 2 and 3, Fig. 1), of which 17 occurred in *C. chengiana *(N = 89), seven in *C. duclouxiana *(N = 86), two in *C. funebris *(N = 89) and four among *C. gigantea *(N = 58), *C. jiangeensis *(N = 1)*, C. austrotibetica *(N = 22), *C. cashmeriana *(N = 4) and *C. torulosa *(N = 22). No intraspecific cpDNA variation was recorded in *C. gigantea, C. jiangeensis*, *C. cashmeriana*, *C. austrotibetica *and *C. torulosa*. *Cupressus cashmeriana *and *C. austrotibetica *shared the same haplotype, which differed by a single nucleotide from the haplotype possessed by *C. torulosa*. These two sequences were very different from the haplotype possessed by *C. gigantea*. A neighbour joining analyses showed that the 30 haplotypes clustered into six different lineages (A-F) with most haplotypes being species-specific (Fig. 2). Although all six lineages were recovered in the 50% strict consensus tree of 51267 most parsimonious trees (CI = 0.77, RI = 0.87, Length = 141), the pattern of relationships between lineages received low bootstrap support (Fig. 2). Lineage A comprised nine haplotypes possessed by individuals of *C. chengiana *sampled from Gansu province, China. In contrast, the other eight haplotypes detected in this species were present only in individuals sampled from Sichuan, China, which together with the single haplotype (H5) recovered from *C. jiangeensis*, comprised lineage E. The sister relationship between lineages A and E was poorly supported. Lineage D was composed of the single haplotype present in *C. torulosa *plus the haplotype shared by *C. austrotibetica *and *C. cashmeriana*, and was sister to lineages A and E (Fig. 2) of *C. chengiana*. The seven different haplotypes detected in *C. duclouxiana *formed a monophyletic lineage C, which was sister to lineage B that consisted of the single haplotype possessed by *C. gigantea*. Lineage F comprised the two haplotypes specific to *C. funebris *and was sister to the five other lineages (A to E) and to two cypress species occurring out of Asia. To evaluate the origins of these lineages (species), we selected the commonest haplotype representing each lineage and dated their origins. According to the estimated nucleotide mutation rate for *Cupressus *(Additional File 4), *C. funebris *was estimated to have diverged from the other lineage/species approximately 26 million years ago (Mya) (Additional File 4). *C. duclouxiana *in the eastern QTP diverged from the western *C. gigantea *around 10 Mya, while the eastern *C. chengiana *diverged from the western *C. torulosa/C. austrotibetica/C. cashmeriana *lineage around 13 Mya. The intraspecific divergence that gave rise to the two different lineages of *C. chengiana *identified was dated to approximately nine Mya.

Because the number of mutations distinguishing any two lineages was always greater than six, we constructed separate haplotype networks for the three lineages that contained most haplotypes (i.e. lineages A, C and E). Within lineage E, two haplotypes H10 and H3 occurred in a small sub-lineage that was separated by four mutations from other haplotypes (Fig. 2). However, H10 co-occurred with the most frequent haplotype in this lineage, H4, in most sampled populations. The single haplotype (H5) recovered from *C. jiangeensis *was also present in lineage E and is derived from H4. In lineage A, H17 was the most distantly related haplotype relative to other haplotypes (Fig. 2).

### Diversity among and within species

Genetic differentiation across all species was high (Table 1, 2) and showed significant phylogeographic structure (*N*_ST _(0.962) > *G*_ST _(0.791), P < 0.05). Mantel tests revealed a significant association (*r*^2 ^= 0.2285, P < 0.001) between genetic differentiation and geographic distances between populations over all species. For *C. chengiana *and *C. duclouxiana*, total intraspecific diversity (*H*_T _= 0.791 and 0.659, respectively) across populations was higher, but not substantially, compared with the average within-population diversity (*H*_S _= 0.536 and 0.443, respectively). Within *C. duclouxiana*, interpopulation variation accounted for a high proportion of intraspecific variation (23.3%, Table 2), indicating strong differentiation between populations. The significant phylogeographic structure of *C. chengiana *reflected the presence of two distinct lineages (A and E) within this species that were located in Gansu and Sichuan provinces, respectively. This regional divergence accounted for 44.2% of the total variation recorded within *C. chengiana *(Table 2). Variation within each of these lineages was almost entirely attributed to variation within populations (Table 2, note that H5 of *C. jiangeensis *was included in the calculations for lineage E). Rare haplotypes were frequent in many populations that were representatives of lineages A or E. In *C. funebris*, two haplotypes were recovered, but no variation was recorded among populations.

**Table 1:** Estimates of average genetic diversity within populations (*H*_S_), total gene diversity (*H*_T_), interpopulation haplotype differentiation (*G*_ST_), and interpopulation haplotype differentiation taking into account sequence difference (*N*_ST_) (mean ± SE in parentheses)

Species	*H*_S_	*H*_T_	*G*_ST_	*N*_ST_	*N*_ST _- *G*_ST_
*C. chengiana*	0.536 (0.079)	0.791 (0.051)	0.322 (0.074)	0.558 (0.056) *	0.236
*C. chengiana *(lineage E, Sichuan populations)	0.471 (0.136)	0.489 (0.117)	0.037 (0.093)	-0.007 (0.075) ^ns^	-0.044
*C. chengiana *(lineage A, Gansu populations)	0.618(0.052)	0.597(0.060)	-0.035(0.053)	-0.022(0.083)^ns^	0.013
*C. funebris*	0.254 (0.060)	0.249 (0.050)	-0.021 (0.055)	-0.021 (0.055) ^ns^	0
*C. duclouxiana*	0.443(0.095)	0.659(0.098)	0.328(0.116)	0.488 (0.092)^ns^	0.160
*C. gigantea*	NC	NC	NC	NC	NC
*C. torulosa*	NC	NC	NC	NC	NC
*C. cashmeriana *& *C. austrotibetica*	NC	NC	NC	NC	NC
*C. torulosa *s.l. *& C. gigantea*	0 (0)	0.665(0.039)	1 (NC)	1 (NC)	0
All species	0.190(0.034)	0.908(0.012)	0.791(0.036)	0.962(0.013)*	0.171

*indicates that *N*_ST _is significantly different from *G*_ST _(0.01 <*P *< 0.05); ns indicates that *N*_ST _is not significantly different from *G*_ST _(*P *> 0.05); NC, not computed due to small sample size; *C. torulosa *s.l. includes *C. torulosa, C. cashmeriana *and *C. austrotibetica*

**Table 2:** Analysis of molecular variance (AMOVA) for cpDNA haplotypes of cypress species

Species/lineage	Source of variation	d.f.	SS	VC	Variation (%)	Fixation index
*C. chengiana *(lineage E, Sichuan populations)	Among populations	5	1.827	0.017	6.8	*F*_ST _= 0.068
	Within populations	44	10.213	0.232	93.2	
	Total	49	12.040	0.249		
*C. chengiana *(lineage A, Gansu populations)	Among populations	3	0.925	-0.001	-0.2	*F*_ST _= -0.002
	Within populations	36	11.325	0.315	100.2	
	Total	39	12. 250	0.314		
*C. chengiana*	Among lineages	1	10.066	0.218	43.9	*F*_CT _= 0.439*
	Among populations within groups	8	2.752	0.009	1.8	*F*_SC _= 0.032
	Within populations	80	21.538	0.269	54.3	*F*_ST _= 0. 457*
	Total	89	34.356	0.496		
*C. funebris*	Among populations	10	0.951	-0.004	-3.5	*F*_ST _= -0.035
	Within populations	78	10.150	0.130	103.5	
	Total	88	11.101	0.126		
*C. duclouxiana*	Among populations	10	6.707	0.061	23.3	*F*_ST _= 0.233*
	Within populations	75	15.142	0.202	76.7	
	Total	85	21.849	0.263		
*C. gigantea*	Among populations	5	0	0	0	*F*_ST _= 0
	Within populations	52	0	0	0	
	Total	57	0	0		
*C. torulosa*	Among populations	11	0.952	0.053	100.0	*F*_ST _= 1.000
	Within populations	9	0	0	0	
	Total	20	0.952	0.053		
*C. cashmeriana &* *C. austrotibetica*	Among populations	7	0	0	0	*F*_ST _= 0
	Within populations	18	0	0	0	
	Total	25	0	0	0	
*C. torulosa *s.l. and *C. gigantea*	Among groups	3	30.659	0.469	97.6	*F*_CT _= 0.976*
	Among populations within groups	22	0.955	0.012	2.4	*F*_SC _= 1.000
	Within populations	80	0	0	0	*F*_ST _= 1.000*
	Total	105	31.613	0.481		
All species	Among species	7	100.946	0.319	63.5	*F*_CT _= 0.635*
	Among populations within species	58	20.258	0.039	7.7	*F*_SC _= 0.210 *
	Within populations	305	44.123	0.145	28.8	*F*_ST _= 0.712 *
	Total	370	165. 326	0.502		

d.f., degrees of freedom; SS, sum of squares; VC, variance components; * P < 0.001.

## Discussion and Conclusions

Two broad findings emerge from our population-level phygeographic study of the eight *Cupressus *species that occur in the QTP and adjacent regions. First, there was generally good correspondence between the major plastid DNA lineages/haplotypes identified and the morphological taxonomy of species in the group [12]. Such divergence in plastid DNA sequence between species in this group probably results from their allopatric origins and the maintenance of broadly allopatric distributions following extensive uplifts of the QTP since the Miocene. Second, the amounts and pattern of intraspecific plastid DNA variation recorded differed greatly among the species examined. Species with restricted natural distributions contained low levels of intraspecific diversity relative to those having wide natural distributions. These two findings are important to an improved understanding of the importance of geographical isolation on the development of high plant species diversity in the QTP biodiversity hotspot and also the distribution of intraspecific diversity within this region.

### Allopatric divergence

Phylogenetic relationships among the major plastid DNA lineages detected were largely consistent with those resolved for species in a previous plastid DNA phylogeny constructed by Little [12] based on other plastid DNA sequences and mainly single sample per species. Interestingly, in both phylogenies the Mediterranean *C. sempervirens *and *C. atlantica *were nested within the Asian lineage indicating that they probably originated from this lineage. Phylogenetic relationships resolved in both plastid DNA phylogenies were a little different from those constructed from RAPD or nuclear ITS sequence data [12,28,29]. Because these latter studies sampled only one or very few individuals from each species, it remains unknown whether the nuclear variation reported was species-specific or not. In contrast to the other studies, our investigation is the only one to have examined molecular variation in cypresses at the population level. Our results suggest that two species (*C. austrotibetica *and *C. cashmeriana*) share the same haplotype, which is very similar to the single haplotype detected in *C. torulosa*. However, all three of these species differ in morphology [12]. The fact that two very distinct cpDNA lineages were recovered from northern and southern populations of *C. chengiana*, is of further interest in that plants from these two regions have sometimes been treated as separate subspecies [13,14]. Thus, our cpDNA data support this division; however, morphological as well as further molecular studies are necessary before a taxonomic revision of this and other Asian cypress species is contemplated.

Our data show that plastid DNA lineage sorting is nearly complete between recognized morphological units of Asian *Cupressus *(Fig. 2). Furthermore, across all populations and species examined, a strong signal for phylogeographic structure is present: *N_ST _* (0.962) was significantly larger than *G_ST _* (0.791, P < 0.05) (Table 1), demonstrating that gene-flow is very low between these broadly allopatric species (Fig. 1). According to preliminary calibrations, *C. funebris *diverged from the other lineage/species around 26 million years ago (Mya), while the two major east-west divergences between *C. duclouxiana *and *C. gigantea*, and between *C. chengiana *and the *C. torulosa/C. austrotibetica/C. cashmeriana *lineage, were dated to around 10 Mya and 13 Mya, respectively. Although these estimates of dates of origin should be treated with caution, they correspond well with geological evidence that the QTP was extensively uplifted from the early Miocene to the Pliocene [30-34]. It is likely that these extensive uplifts created fragmentation and isolation of habitats, which promoted repeated allopatric speciation of *Cupressus *in the QTP and adjacent regions. The formation of high mountains separated by deep valleys will have reduced gene flow between *Cupressus *populations that were diverging into different species in different valleys and hastened the fixation of locally specific haplotypes. However, allopatric differentiation usually results in the origin of 'paraphyletic' species because of incomplete lineage sorting [6]. Thus, the formation of almost completely mutual 'monophyletic lineages' correlated with morphological units, which we recorded here, is likely to have been brought about by repeated extinctions and/or genetic drift in the isolated habitats [3-5]. A hypothesis of the allopatric origin of each *Cupressus *species in a different valley or valley system is supported by the low level of inter-population differentiation and minimal population substructure observed in most species/lineages except *C. duclouxiana *and *C. chengiana *(Table 2). In these latter two species, a high level of population differentiation was recorded among geographically isolated regions, but very little population divergence within such regions (Fig. 1, Table 2). Two lineages of *C. chengiana *may partly reflect a long history of divergence with the species fragmented into two separate areas in the north and south of western China approximately nine Mya.

The presence of strong geographical isolating barriers in the form of high mountains might also have contributed to rapid species diversification in other plant groups present in the QTP [9,35-38]. For example, in the genus *Ligularia*, most species with distinct morphologies and restricted distributions are thought to have originated through allopatric isolation from a common ancestor within a very short time correlated with the period of extensive uplift of the QTP [36]. Our phylogeographic analysis at the population level of closely related cypress species in the QTP, thus provides further evidence for geographic isolation acting as a driving force of high rates of speciation in this and adjacent regions.

### Intraspecific diversity

Whereas high levels of plastid DNA diversity were recorded in *C. chengiana *and *C. duclouxiana*, only one or two haplotypes were present in each of *C. gigantea*, *C. austrotibetica*, *C. cashmeriana*, *C. torulosa *and *C. funebris*. This difference in intraspecific diversity is highly correlated with the natural distribution ranges of the sampled species [10-19]. *Cupressus gigantea*, *C. austrotibetica*, *C. cashmeriana *and *C. torulosa *occur sparsely at high altitude in the QTP and the west Himalayas, which have been highly vulnerable to the effects of global climatic change [30]. These species might have been more widespread during periods of more favorable climate in their history. Alternatively or in addition, their restricted distributions might be a consequence of the negative impacts of human activities. Either way, they may have experienced severe population bottlenecks in the relatively recent past which reduced significantly their genetic diversity. In contrast to the other four species, *Cupressus funebris *is distributed in central and eastern China and adjacent regions where adverse climatic effects should have been less intense. It seems, however, that most natural populations of this species have become extinct in the wild and only a few, comprising trees of large size, survived in south China and north Vietnam [10-19]. However, according to our own field observations, even these populations (for example, the Ruyuan population in Guangdong) (Additional File 1) are likely to have been artificially cultivated because these large trees grow around one ancient temple. The presence of only two haplotypes in this species may be a legacy of its widespread cultivation and eradication from the wild as seems also to be the case for *C. sempervirens *in Europe [39]. In contrast to these Asian cypress species showing low levels of plastid DNA diversity, *C. duclouxiana*, and *C. chengiana *contain high levels of diversity and occur at lower altitude at the eastern margin of the QTP, where they might have experienced fewer bottlenecks and/or founder effects caused by historical climatic change [30]. It is also to be noted that most extant populations of *C. chengiana *are natural, as are populations of *C*. *duclouxiana *except those cultivated in North Yunnan, and therefore are likely to have been less affected by human activities and subsequent bottleneck effects.

All of the species examined, except for *C. duclouxiana *and *C. funebris*, are listed as endangered [11]. Genetic diversity is critically important for a species' evolutionary potential in the face of environmental change, and loss of genetic diversity is often associated with reduced fitness [40]. The amounts and distribution of genetic diversity within these cypress species are thus important in regard to formulating appropriate plans for conservation purposes. Based on the amounts and distribution of plastid DNA sequence variation within these species, it would seem that for most species, conservation of only a few populations will be sufficient to maintain plastid DNA diversity at current levels. However, the high population differentiation found in *C. duclouxiana *(Table 1) suggests a need to conserve more populations for maintenance of plastid DNA diversity in this species. Similarly, in *C. chengiana *our results make clear that conservation of multiple populations in both Gansu and Sichuan provinces will be necessary to ensure that plastid DNA diversity is maintained in this species.

## Methods

### Species and samples

Leaf samples were collected from eight Asian *Cupressus *species that were identified according to the recent taxonomic revision of Little [12]: *C. chengiana *S. Y. Hu, *C. jiangeensis *N. Zhao, *C. cashmeriana *Royle ex Carrière, *C. duclouxiana *Hickel ex Camus, *C. funebris *Endl., *C. torulosa *D. Don, *C. austrotibetica *Silba and *C. gigantea *W. C. Cheng & L. K. Fu. The one Asian species not included was *C. tonkinensis *Silba, which occurs in Vietnam and across the Vietnam-China border. In total, samples were collected from 371 trees over 66 populations (Additional File 1), which covered most of the natural or cultivated distribution of the species examined. All individuals of *C. austrotibetica *sampled appeared to have been artificially cultivated because they grew around ancient temples, while three populations of *C. duclouxiana *(29, 30 and 31) entirely comprised cultivated trees. Most populations of *C. chengiana *and *C. gigantea *were natural, although a few individuals in the Lixian seemed to have been cultivated. Only one individual of *C. jiangeensis *was available for analysis. When more than one sample was collected from a population, samples were taken from trees at least 50 m apart. The latitude, longitude and altitude of localities for most populations sampled were recorded using an Etrex GIS monitor (Garmin, Taiwan). Also surveyed were three cultivated individuals of *C. sempervirens *sampled from England, Italy and China, respectively, and one individual of *C. atlantica*. We sequenced three individuals of the New World cypress species as outgroups because recent phylogenetic analyses suggest that all Old World cypress species comprise a monophyletic lineage that is distinct from the New World cypress lineage and juniper lineage [12][41].

### DNA extraction, amplification and sequencing

Genomic DNA was isolated from approximately 50 mg of silica-gel dried, leaf-needle material using the CTAB method [42]. Three to five individuals from each of 25 populations sampled across most species were used in an initial screen of plastid DNA polymorphisms. Three different plastid DNA primer pairs were selected for use in this scan, and all revealed polymorphism. Subsequently, we used these three primer pairs to survey all individuals sampled for the *trn*D-*trn*T [43], *trn*S-*trn*G [44] and *trn*L-*trn*F [45] regions, respectively. Polymerase chain reaction (PCR) was performed following the procedure of Zhang *et al*. [46] in a 25-μL volume, containing 10-40 ng plant DNA, 50 mM Tris-HCl (pH 8.0), 1.5 mM MgCl_2_, 0.5 mM dNTPs, 2 μM of each primer, and 0.75 unit of Taq polymerase.

PCR products were purified using a TIANquick Midi Purification Kit following the recommended protocol (TIANGEN). Sequencing reactions were performed with the PCR primers described above, to cover the whole PCR segment using ABI Prism BigdyeTM Terminator Cycle Sequencing Ready Reaction Kit. Sequencing was performed with BigDye Terminator 3.1 (Applied Biosystems) on an ABI PRISM 3130×l sequencer using the same primers as used for PCR amplifications. Sequences were aligned with CLUSTAL X [47] and double-checked manually. A matrix of combined sequences was constructed for the 371 individuals examined, and showed that 30 different cpDNA sequences (haplotypes) were present. All sequences have been deposited in the GenBank databases under accession numbers HM008329-HM008379.

### Data analysis

We used PAUP 4.0 [48] to perform maximum parsimony and neighbour joining analyses to examine phylogenetic relationships among cpDNA haplotypes (Additional File 3). All indels were coded as binary characters (0 or 1) using the GAPCODER program [49] and those with further indels were sequentially assumed as multiple evolutionary events. Maximum parsimony analyses (equally weighted characters and nucleotide transformations) involved a heuristic search strategy with 100 replicates of random addition of sequences, in combination with ACCTRAN character optimization and MULPARS+TBR branch-swapping and STEEPEST DESCENT options on. Bootstrap values for MP analyses (1000 replicates) were used to assess the relative support for monophyletic groups [50].

We used BEAST (under the common model GTR) [51] to estimate the mutation rate of *Cupressus *and the possible divergence timescales of the main cpDNA lineages across the eight Asian cypress species. We edited two cpDNA (*trn*D-*trn*T, *trn*S-*trn*G and *trn*L-*trn*F) datasets respectively. The first comprised three *Cupressus *species, three *Juniperus *species, three *Calitropsis *species, one species for *Platycladus*, *Calocedrus*, *Chamaecyparis *and *Thuja*. The other included the most common haplotypes of the *Cupressus *species and three *Calitropsis *species. For analyses of both datasets, we excluded all indels and only nucleotide mutations were considered. A strict molecular clock assumption was rejected for both datasets because of significant rate heterogeneity among lineages. We used BEAST version 1.5.3 employing a Bayesian Markov chain Monte Carlo (MCMC) chain and co-estimated topology to estimate the possible mutation rate of *Cupressus *and divergence timescales across the Asian cypress species. Under the GTR model of nucleotide substitution with a gamma distribution and four rate categories, three tree prior models (Coalescent: constant size, exponential growth; and Speciation: Yule process) were implemented with rate variation across branches assumed to be uncorrelated and log-normally distributed. The model that yielded the highest posterior probability estimates was employed to perform a final analysis. For all analyses, posterior distributions of parameters were approximated using two independent MCMC analyses of 100,000,000 generations with 20% burn-in. The program Tracer v1.5 [51] was used to check Effective Sample Size (ESS) and the program TreeAnnotator 1.5.3 (part of the BEAST 1.5.3 package) was used to combine all samples and to converge and/or summarize output results. A tree with ages for each node and their 95% HPD were displayed and modified in FigTree v1.3.1 [51]. According to the fossil calibrations of the first dataset, a mutation rate of cpDNA (3.2 × 10^-10 ^substitutions per site per year) was obtained (Additional File 4). This mutation rate was used to calibrate the interspecific or interlineage divergences across the cypress species (Additional File 4).

An examination of interrelationships between haplotypes within lineages recovered in phylogenetic trees was conducted using TCS 1.21 [52]. TCS was run with the default parsimony connection limit of 95%. We assumed that all coded indels and mutations evolved at an equal rate. Estimates of unbiased genetic diversity (*H*_E_) [equivalent to expected heterozygosity for diploid data] were calculated as ś: ś = n/(n-1) (1- p*_i_*^2^), in which n is the number of samples in the population, k is the number of haplotypes, and p is the population frequency of the *i*th haplotype [53]. Where there were insufficient numbers of individuals in a population, multiple nearby populations were combined into a larger one for analysis (for example, populations 43-46, each comprising a single tree, were treated together as one population). Average gene diversity within populations (*H*_S_), total gene diversity (*H*_T_) and two measures of population differentiation, *G*_ST _(coefficient of genetic variation over all populations [54]) and *N*_ST _(equivalent coefficient taking into account sequence similarities between haplotypes) were calculated using the program PERMUT (available at http://www.pierroton.inra.fr/genetics/labo/Software/) [55]. Significant phylogeographic structure was inferred by testing whether *N*_ST _was significantly greater than *G*_ST _using PERMUT with 1000 permutations. Genetic structure was further examined by analysis of molecular variance (AMOVA, [56]) as implemented in Arlequin version 3.01 [57]. The significance of isolation by distance between populations was tested using a Mantel test with 1000 random permutations on matrices of pairwise population *F*_ST _values and the natural logarithm of geographical distances [58]. Pairwise *F*_ST _values between populations were estimated using Arlequin while geographical distances between populations were calculated with the help of the program at http://www.indo.com/distance/ (e.g. [59]).

## Authors' contributions

JQL designed the study. JQL, GM and ZXCR collected materials. TTX and GLW finished molecular experiments. TTX, KSM and FKD analyzed data. JQL, RJA, RIM and TTX wrote the manuscript. All authors read and approved the final manuscript.

## Supplementary Material

Additional File 1 **Locations of sites and voucher specimens of eight Asian *Cupressus *species surveyed for cpDNA variation**. Click here for file

Additional File 2 **Haplotype frequencies and genetic diversity (*H*_E_) of populations of eight Asian *Cupressus *species surveyed for plastid DNA variation**. Click here for file

Additional File 3 **Variable sites for aligned sequences of three plastid DNA fragments among 30 haplotypes resolved in eight Asian *Cupressus *species (-, one indel)**. Sequences are numbered from the 5' to the 3'end in each region. Click here for file

Additional File 4 **Estimates of mutation rate for *Cupressus *and dates of divergence between the Asian species/lineage estimated by BEAST**. Click here for file
