# Supplementary material for: Phylogeography and allopatric divergence of cypress species (Cupressus L.) in the Qinghai-Tibetan Plateau and adjacent regions
Source: BMC Evol Biol. 2010 Jun 22;10:194. doi: 10.1186/1471-2148-10-194 (PMC3020627; doi:10.1186/1471-2148-10-194)
Supplement: Additional File 1 — Locations of sites and voucher specimens of eight Asian Cupressus species surveyed for cpDNA variation. [file 1471-2148-10-194-S1.DOC]

**Additional File 1** Locations of sites and voucher specimens of eight Asian *Cupressus* species surveyed for plastid DNA variation

| P | Taxa | Population/ | Latitude | Longitude | Altitude | Cultivated | Specimens | | |
| --- | --- | --- | --- | --- | --- | --- | --- | --- | --- |
|  |  | Location | (N) | (E) | (m) |  | Collection number | Collector | Place of deposition |
| 1 | *C. chengiana*  (Sichuan locations) | Lixian, SC | 31°30.00' | 102°56.00' | 1600-2100 | No | JQ Liu 1913 | Jianquan Liu | Lanzhou university, China |
| 2 |  | Lixian, SC | 31°24.54' | 103°06.92' | 1954 | No | TB-07022 | Jianquan Liu | Lanzhou university, China |
| 3 |  | Jinchuan, SC | 31°47.46' | 101°56.48' | 2470-2400 | No | JQ Liu 1995 | Jianquan Liu | Lanzhou university, China |
| 4 |  | Xiaojin, SC | 30°32.00' | 101°35.00' | 3780 | No | JQ Liu 2406 | Jianquan Liu | Lanzhou university, China |
| 5 |  | Danba, SC | 30°07.84' | 102°10.43' | 1680 | No | JQ Liu 2691 | Jianquan Liu | Lanzhou university, China |
| 6 |  | Markan,SC | 31°55.72' | 102°02.02' | 2417 | No | TB-07029 | Jianquan Liu | Lanzhou university, China |
| 7 | *C. jiangensis* | Jiange, SC* | 32°01.00' | 105°28.00' | 535 | No | JQ Liu 2732 | Jianquan Liu | Lanzhou university, China |
| 8 | *C. chengiana*  (Gansu locations) | Wenxian, GS | 32°44.47' | 104°54.45' | 888 | No | JQ Liu 2736 | Jianquan Liu | Lanzhou university, China |
| 9 |  | Wudu, GS | 33°14.90' | 104°59.15' | 1400 | No | JQ Liu2005016 | Jianquan Liu | Lanzhou university, China |
| 10 |  | Wenxian, GS | 33°12.03' | 105°02.13' | 1025 | No | TB-07014 | Jianquan Liu | Lanzhou university, China |
| 11 |  | Zhouqu,GS | 33°52.27' | 104°08.59' | 1531 | No | JQ Liu2005007 | Jianquan Liu | Lanzhou university, China |
| 12 | C. funebris | Kangxian, GS* | 33°20.00' | 105°32.00' | 2400 | Yes | JQ Liu2005003 | Jianquan Liu | Lanzhou university, China |
| 13 |  | Jiange, SC* | 32°14.13' | 105°33.17' | 617 | Yes | JQ Liu2005006 | Jianquan Liu | Lanzhou university, China |
| 14 |  | Beichuan, SC* | 31°50.26' | 104°15.35' | 777 | Yes | JQ Liu2699 | Jianquan Liu | Lanzhou university, China |
| 15 |  | Wenchuan, SC* | 31°03.71' | 103°29.18' | 1100 | Yes | JQ Liu05111 | Jianquan Liu | Lanzhou university, China |
| 16 |  | Mianyang, SC* | 31°32.57' | 104°48.99' | 523 | Yes | TB-07067 | Jianquan Liu | Lanzhou university, China |
| 17 |  | Zitong, SC* | 31°39.59' | 105°14.21' | 493 | Yes | TB-07068 | Jianquan Liu | Lanzhou university, China |
| 18 |  | Guangyuan, SC* | 32°37.00' | 105°52.36' | 652 | Yes | TB-07069 | Jianquan Liu | Lanzhou university, China |
| 19 |  | Jinyan, SC* | 29°40.02' | 104°03.50' | 419 | Yes | TB-07073 | Jianquan Liu | Lanzhou university, China |
| 20 |  | Chongqing, CQ* | 29°33.06' | 106°27.00' | 300 | Yes | JQ Liu2008-JY | Jianquan Liu | Lanzhou university, China |
| 21 |  | Shennongjia, HB* | 31°21.06' | 110°18.06' | 1700 | Yes | JQ Liu2008-CQ | Jianquan Liu | Lanzhou university, China |
| 22 |  | Ruyuan, GD* | 24°59.34' | 113°09.03' | 411 | Yes | JQ Liu2008-SNJ | Jianquan Liu | Lanzhou university, China |
| 23 | C. gigantea | Jiacha, XZ | 29°02.29' | 093°03.23' | 3130-3430 | No | TT-07020 | Jianquan Liu | Lanzhou university, China |
| 24 |  | Langxian, XZ | 28°59.95' | 093°14.11' | 3060 | No | JQ Liu 1170 | Jianquan Liu | Lanzhou university, China |
| 25 |  | Milin, XZ | 29°20.40' | 094°22.63' | 2950 | No | JQ Liu 1171 | Jianquan Liu | Lanzhou university, China |
| 26 |  | Linzhi, XZ | 29°40.00' | 094°20.00' | 3040 | No | JQ Liu 1183 | Jianquan Liu | Lanzhou university, China |
| 27 |  | Langxian, XZ | 29°08.63' | 093°27.64' | 3020 | No | JQ Liu 1191 | Jianquan Liu | Lanzhou university, China |
| 28 |  | Milin, XZ | 29°07.59' | 093°50.93' | 3050 | No | JQ Liu 2626 | Jianquan Liu | Lanzhou university, China |

**Additional File 1** continued

| P | Taxa | Population/ | Latitude | Longitude | Altitude | Cultivated | Voucher specimens | | |
| --- | --- | --- | --- | --- | --- | --- | --- | --- | --- |
|  |  | Location | (N) | (E) | (m) |  | Collection number | Collector | Place of deposition |
| 29 | *C. duclouxiana* | Kunming, YN* | 25° 01.00' | 102°41.00' | 1990 | Yes | JQ Liu 2154 | Jianquan Liu | Lanzhou University, China |
| 30 |  | Kunming, YN* | 25° 03.00' | 102°43.08' | 1930 | Yes | JQ Liu 2162 | Jianquan Liu | Lanzhou University, China |
| 31 |  | Kunming, YN* | 25° 15.17' | 102°44.46' | 1957 | Yes | TB-07059 | Jianquan Liu | Lanzhou University, China |
| 32 |  | Lufeng, YN | 25° 05.82' | 101°48.26' | 1801 | Yes | TB-07058 | Jianquan Liu | Lanzhou University, China |
| 33 |  | Eryuan, YN | 26°14.68' | 099°56.49' | 2100 | Yes | TB-07057 | Jianquan Liu | Lanzhou University, China |
| 34 |  | Binchuan,YN | 25°56.68' | 100°24.03' | 2203 | Yes | TB-07056 | Jianquan Liu | Lanzhou University, China |
| 35 |  | Yongsheng, YN | 26°44.39' | 100°45.96' | 2170 | Yes | JQ Liu 05038 | Jianquan Liu | Lanzhou University, China |
| 36 |  | Yulong, YN | 26°56.19' | 099°57.07' | 1830 | Yes | JQ Liu 05048 | Jianquan Liu | Lanzhou University, China |
| 37 |  | Lijiang, YN | 27°08.28' | 100°14.17' | 2800 | Yes | JQ Liu 2290 | Jianquan Liu | Lanzhou University, China |
| 38 |  | Lijiang, YN | 27°07.80' | 100°14.40' | 2900 | Yes | JQ Liu 05052 | Jianquan Liu | Lanzhou University, China |
| 39 |  | Xianggelila, YN | 27°20.17' | 099°57.82' | 2510 | No | JQ Liu 05060 | Jianquan Liu | Lanzhou University, China |
| 40 |  | Zhongdian, YN | 28°07.00' | 099°27.00' | 2780 | No | 07-24-01 | Georg Miehe & Sunzhehua | Lanzhou University, China |
| 41 |  | Bennzilan,YN | 28°08.43' | 099°26.92' | 2559 | No | TB-07051 | Jianquan Liu | Lanzhou University, China |
| 42 |  | Daocheng, SC | 28°23.05' | 100°14.35' | 2752 | No | TB-07044 | Jianquan Liu | Lanzhou University, China |
| 43 |  | Deqin, YN | 28°33.00' | 098°50.00' | 2600 | No | 07-93-01 | Georg Miehe & Sunzhehua | Lanzhou University, China |
| 44 |  | Deqin, YN | 28°22.27' | 099°03.41' | 2870 | No | 04-99-10 | Georg Miehe et al. | University of Marburg, Germany |
| 45 |  | Deqin, YN | 28°21.57' | 098°53.55' | 4400 | No | JQ Liu 05087 | Jianquan Liu | Lanzhou university, China |
| 46 |  | Mangkang, XZ | 29°41.01' | 098°34.54' | 2510 | No | JQ Liu 2352 | Jianquan Liu | Lanzhou university, China |
| 47 | *C. austrotebitica* | Tongmai, XZ* | 30°01.67' | 095°16.75' | 2600 | Yes | LO-04-74 & 04-74 -01 | Lars & Miehe | University of Marburg, Germany |
| 48 |  | Yigong, XZ* | 30°08.44' | 095°01.05' | 2152 | Yes | 07-038-01 | Sabine Miehe | Lanzhou university, China |
| 49 |  | Yigong, XZ* | 30°01.00' | 094°58.00' | 2300 | Yes | ljq07274 | Jianquan Liu | Lanzhou university, China |
| 50 |  | Bomi, XZ* | 30°08.38' | 095°01.04' | 2400 | Yes | Bomi | Zhaxiciren | Tibet University, |
| 51 | *C. cashmeriana* | Bhutan | 27°30.00' | 089°20.00' | 2500 | Yes | 00-143-03 | Georg Miehe *et al.* | University of Marburg, Germany |
| 52 |  | Bhutan | 27°24.00' | 089°56.00’ | 2500-2640 | Yes | 99-214-06 | Georg Miehe *et al.* | University of Marburg, Germany |
| 53 |  | Bhutan | 27°37.00' | 090°10.00’ | 2650 | Yes | 99-226-01 | Georg Miehe *et al.* | University of Marburg, Germany |
| 54 |  | Bhutan | 27°35.00' | 089°58.00’ | 2630 | Yes | Britain19696979 | Georg Miehe *et al.* | University of Marburg, Germany |

**Additional File 1**  continued

| P | Taxa | Population/ | Latitude | Longitude | Altitude | Cultivated | Voucher specimens | | |
| --- | --- | --- | --- | --- | --- | --- | --- | --- | --- |
|  |  | Location | (N) | (E) | (m) |  | Collection number | Collector | Place of deposition |
| 55 | *C. torulosa* | Dolpo, Nepal | 29°34.00' | 082°15.00' | 2050 | Yes | 99-21-01 | Georg Miehe *et al.* | University of Marburg, Germany |
| 56 |  | Dolpo, Nepal | 29°35.00' | 082°26.00' | 2400 | Yes | 99-23-01 | Georg Miehe *et al.* | University of Marburg, Germany |
| 57 |  | Dolpo, Nepal | 29°34.30' | 082°18.00' | 2300 | Yes | TB07020 | Georg Miehe *et al.* | University of Marburg, Germany |
| 58 |  | Lower Dolpo, Nepal | 29°07.00' | 082°55.00' | 3250 | Yes | 99-88-(03,04,05) | Georg Miehe *et al.* | University of Marburg, Germany |
| 59 |  | Lower Dolpo, Nepal | 29°07.00' | 082°53.00' | 3000 | Yes | 99-89-(01,02,03,04,05,06) | Georg Miehe *et al.* | University of Marburg, Germany |
| 60 |  | Lower Dolpo, Nepal | 29°08.00' | 082°53.00' | 2750 | Yes | 99-90-(01,02) | Georg Miehe *et al.* | University of Marburg, Germany |
| 61 |  | Lower Dolpo, Nepal | 29°00.00' | 082°53.00' | 2200 | Yes | 99-92-01 | Georg Miehe *et al.* | University of Marburg, Germany |
| 62 |  | Lower Dolpo, Nepal | 29°10.00' | 082°56.00' | 3650 | Yes | 99-93-01 | Georg Miehe *et al.* | University of Marburg, Germany |
| 63 |  | Mustang, Nepal | 28°54.00' | 083°46.00' | 3670 | Yes | 01-086-01,01-089-01 | Georg Miehe *et al.* | University of Marburg, Germany |
| 64 |  | Mustang, Nepal | 28°54.00' | 083°47.00' | 3300 | Yes | 01-087-01 | Georg Miehe *et al.* | University of Marburg, Germany |
| 65 |  | Mustang, Nepal | 28°53.00' | 083°46.00' | 3160 | Yes | 01-101-02 | Georg Miehe *et al.* | University of Marburg, Germany |
| 66 |  | Mustang, Nepal | 28°54.00' | 083°45.00' | 3410 | Yes | 01-119-01 | Georg Miehe *et al.* | University of Marburg, Germany |

SC: Sichuan; GS, Gansu; YN, Yunnan; HB, Hubei; GD, Guangdong; XZ, Xizang (Tibet); CQ, Chongqing. * indicate that the collected populations are likely to be artificially cultivated according to their habitats, the accompanying species and information for the local inhabitants and most of these populations grow around the ancient temples.
