# Supplementary material for: Phylogeography and allopatric divergence of cypress species (Cupressus L.) in the Qinghai-Tibetan Plateau and adjacent regions
Source: BMC Evol Biol. 2010 Jun 22;10:194. doi: 10.1186/1471-2148-10-194 (PMC3020627; doi:10.1186/1471-2148-10-194)
Supplement: Additional File 3 — Variable sites for aligned sequences of three plastid DNA fragments among 30 haplotypes resolved in eight Asian Cupressus species (-, one indel). Sequences are numbered from the 5' to the 3'end in each region. [file 1471-2148-10-194-S3.DOC]

**Additional File 3** Variable sites of aligned sequences of three plastid DNA fragments among 30 haplotypes resolved in eight Asian *Cupressus* species (-, one indel). Sequences are numbered from the 5’ to the 3’end in each region.

| Nucleotide position  Haplotype | 33 | 47 | 49 | 51–114 | 137 | 149 | 153^C^ | 157 | 170 | 230–233 | 559 | 566 | 581 | 595 | 630 | 657 |  | 686 | 753 | 891 | 921 | 951 | 964 | 965–966 | 967–968 | 969 | 970 | 995 | 1021 | 1170 | 1175–1177 | 1189–1213 | 1283–1289 | 1294–1416 | 1417 | 1418–1442 | 1453-1475 |  | 1551 | 1599 | 1632 | 1688 | 1817 | 1863 | 1901 | 1902 | 1903-1963 | 1964-2038 | 2039-2048 | 2049-2070 | 2087 | 2106 | 2153-2158 |
| --- | --- | --- | --- | --- | --- | --- | --- | --- | --- | --- | --- | --- | --- | --- | --- | --- | --- | --- | --- | --- | --- | --- | --- | --- | --- | --- | --- | --- | --- | --- | --- | --- | --- | --- | --- | --- | --- | --- | --- | --- | --- | --- | --- | --- | --- | --- | --- | --- | --- | --- | --- | --- | --- |
|  | *trn*D-T | | | | | | | | | | | | | | | |  | *trn*S-G | | | | | | | | | | | | | | | | | | | |  | *trn*L-F | | | | | | | | | | | | | | |
| 1 | A | C | T | – | C | T | A | C | C | β | C | G | G | C | T | – |  | A | A | A | A | C | – | –– | –– | – | – | A | C | A | TAA | – | – | ω | G | ○ | – |  | C | C | G | A | A | T | – | – | – | – | – | – | A | T | # |
| 2 | A | C | T | – | C | T | A | C | C | β | C | G | G | C | T | – |  | C | A | A | A | C | – | –– | –– | – | – | A | C | A | TAA | – | – | ω | G | ○ | – |  | C | C | G | A | A | T | – | – | – | – | – | – | A | T | # |
| 3 | C | T | C | – | A | T | – | C | T | β | C | T | G | C | C | – |  | C | G | – | – | C | – | –– | –– | T | A | – | C | C | ––– | – | ■ | ω | G | ○ | – |  | C | C | G | G | A | – | – | – | – | – | – | – | A | T | # |
| 4 | C | T | C | – | A | T | – | C | T | β | C | T | G | C | C | – |  | C | G | – | – | C | – | –– | –– | T | A | – | C | C | ––– | – | ■ | ω | G | ○ | – |  | C | C | G | G | A | – | T | G | ◆ | – | – | * | A | T | # |
| 5 | C | T | C | – | A | T | – | C | T | β | C | T | G | C | C | – |  | C | G | – | – | T | – | –– | –– | T | A | – | C | C | ––– | – | ■ | ω | G | ○ | – |  | C | C | G | G | A | – | T | G | ◆ | – | – | * | A | T | # |
| 6 | C | T | C | – | A | T | – | C | T | β | C | T | G | C | C | – |  | C | G | – | – | C | – | TA | TA | T | A | – | C | C | ––– | – | ■ | ω | G | ○ | ▲ |  | C | C | G | G | A | – | T | G | ◆ | – | – | * | A | T | # |
| 7 | C | T | C | – | A | T | – | C | T | β | C | T | G | C | C | – |  | C | G | – | – | C | – | –– | –– | T | A | – | C | C | ––– | – | ■ | ω | G | ○ | – |  | G | C | G | G | A | – | T | G | ◆ | – | – | * | A | T | # |
| 8 | C | T | C | – | A | T | – | C | T | β | C | T | G | C | C | – |  | C | G | – | – | C | – | –– | –– | T | A | – | C | C | ––– | – | ■ | ω | G | ○ | – |  | C | C | G | G | A | – | T | G | ◆ | ★ | ☆ | * | A | T | # |
| 9 | C | T | C | – | A | T | – | C | T | β | C | T | G | C | C | – |  | C | G | – | – | C | – | –– | –– | T | A | – | G | C | ––– | – | ■ | ω | G | ○ | – |  | C | C | G | G | A | – | T | G | ◆ | – | – | * | A | T | # |
| 10 | C | T | C | – | A | T | – | C | T | β | C | G | G | C | C | – |  | C | G | – | – | C | – | –– | –– | T | A | – | C | C | ––– | – | ■ | ω | G | ○ | – |  | C | C | G | G | A | – | – | – | – | – | – | – | A | T | # |
| 11 | C | T | C | – | A | T | – | C | T | β | C | T | G | C | C | – |  | C | G | – | – | C | – | –– | –– | T | A | – | G | C | ––– | – | ■ | ω | G | ○ | – |  | C | C | G | G | A | – | T | G | ◆ | ★ | ☆ | * | A | T | # |
| 12 | C | T | C | – | A | T | – | A | T | β | C | T | G | C | C | – |  | C | G | – | – | C | – | –– | TA | G | A | – | C | C | ––– | δ | ■ | – | – | – | – |  | C | C | G | G | A | – | T | T | ◆ | – | – | * | A | T | # |
| 13 | C | T | C | – | A | T | – | C | T | β | C | T | G | C | C | – |  | C | G | – | – | C | – | –– | TA | T | A | – | C | C | ––– | – | ■ | – | – | – | – |  | C | C | G | G | A | – | T | T | ◆ | – | – | * | A | T | # |
| 14 | C | T | C | α | A | T | – | C | T | β | C | T | G | C | C | – |  | C | G | – | – | C | – | –– | TA | G | A | – | C | C | ––– | – | ■ | – | – | – | – |  | C | C | G | G | A | – | T | T | ◆ | – | – | * | A | T | # |
| 15 | C | T | C | α | A | T | – | C | T | β | C | T | G | C | C | – |  | C | G | – | – | C | – | –– | TA | T | A | – | C | C | ––– | – | ■ | – | – | – | – |  | C | C | G | G | A | – | T | T | ◆ | – | – | * | A | T | # |
| 16 | C | T | C | – | A | T | – | C | T | β | C | G | G | C | C | – |  | C | G | – | – | C | – | –– | TA | G | A | – | C | C | ––– | – | ■ | – | – | – | – |  | C | C | G | G | A | – | T | T | ◆ | – | ☆ | * | A | T | # |
| 17 | C | T | C | – | A | T | – | C | T | β | C | T | G | C | C | – |  | C | G | – | – | C | – | –– | TA | G | A | – | C | C | ––– | – | ■ | – | – | – | – |  | C | C | G | G | A | – | – | – | – | – | – | – | A | T | # |
| 18 | C | T | C | – | A | T | – | C | T | β | C | T | G | C | C | – |  | C | G | – | – | C | – | –– | TA | G | A | – | C | C | ––– | – | ■ | – | – | – | – |  | C | C | G | G | A | – | T | T | ◆ | – | – | * | A | T | # |
| 19 | C | T | C | – | A | T | – | C | T | – | C | T | G | C | C | – |  | C | G | – | – | C | – | –– | TA | G | A | – | C | C | ––– | – | ■ | – | – | – | – |  | C | C | G | G | A | – | T | T | ◆ | – | – | * | A | T | # |
| 20 | C | T | C | – | A | T | – | C | T | β | C | G | G | C | C | – |  | C | G | – | – | C | – | –– | TA | T | A | – | C | C | ––– | – | ■ | – | – | – | – |  | C | C | G | G | A | – | T | T | ◆ | – | – | * | A | T | # |
| 21 | C | T | C | – | A | G | – | C | T | β | C | G | G | C | C | T |  | C | G | – | – | C | A | TA | TA | T | A | – | C | C | ––– | – | ■ | ω | C | ○ | – |  | C | C | G | A | C | – | – | – | – | – | – | – | A | T | # |
| 22 | C | T | C | – | A | G | – | C | T | β | C | G | G | C | C | T |  | C | G | – | – | C | A | TA | TA | T | A | – | C | C | ––– | – | ■ | ω | C | ○ | – |  | C | C | G | A | C | – | – | – | – | – | – | – | T | T | # |
| 23 | C | T | C | – | A | G | – | C | T | β | C | G | G | C | C | T |  | C | G | – | – | C | A | TA | TA | T | A | – | C | C | ––– | – | ■ | ω | C | ○ | – |  | C | A | G | A | C | – | – | – | – | – | – | – | A | T | # |
| 24 | C | T | C | – | A | G | – | C | T | β | C | G | G | C | C | T |  | A | G | – | – | C | A | TA | TA | T | A | – | C | C | ––– | – | ■ | ω | C | ○ | – |  | C | C | G | A | C | – | – | – | – | – | – | – | A | T | # |
| 25 | C | T | C | – | C | G | – | C | T | β | C | T | G | C | C | T |  | C | G | – | – | C | A | TA | TA | T | A | – | C | C | ––– | – | ■ | ω | C | ○ | – |  | C | A | G | A | C | – | – | – | – | – | – | – | A | T | # |
| 26 | C | T | C | – | A | T | – | C | T | β | C | T | G | C | C | T |  | C | G | – | – | C | A | TA | TA | T | A | – | C | C | ––– | – | ■ | ω | C | ○ | – |  | C | A | G | A | C | – | – | – | – | – | – | – | A | T | # |
| 27 | C | T | C | α | A | T | – | C | T | β | C | T | G | C | C | T |  | C | G | – | – | C | A | TA | TA | T | A | – | C | C | ––– | – | ■ | ω | C | ○ | – |  | C | A | G | A | C | – | – | – | – | – | – | – | A | T | # |
| 28 | C | T | C | – | C | G | A | C | T | β | C | T | G | A | C | – |  | C | G | – | – | C | – | TA | TA | T | A | – | C | C | ––– | – | ■ | ω | G | ○ | ▲ |  | C | A | G | A | A | – | – | – | – | – | – | – | A | T | # |
| 29 | C | T | C | – | A | T | – | C | T | β | A | T | A | C | C | – |  | C | G | – | – | C | – | –– | TA | T | A | – | C | C | ––– | – | ■ | ω | G | ○ | – |  | C | C | T | A | A | – | – | – | – | – | – | – | A | T | – |
| 30 | C | T | C | – | A | T | – | C | T | β | A | T | A | C | C | – |  | C | G | – | – | C | – | –– | TA | T | A | – | C | C | ––– | – | ■ | ω | G | ○ | – |  | C | C | T | A | A | – | – | – | – | – | – | – | A | A | – |
|  | | | | | | | | | | | | | | | | | | | | | | | | | | | | | | | | | | | | | | | | | | | | | | | | | | | | | |
| Binary coding |  |  |  | 0 |  |  | 0 |  |  | 0 |  |  |  |  |  | 0 |  |  |  | 0 | 0 |  | 1???  0 | | | | | 0 |  |  | 0 | 0 | 0 | 1? | | | 0 |  |  |  |  |  |  | 0 | 1?? | | | | | |  |  | 0 |
|  |  |  |  | 1 |  |  | 1 |  |  | 1 |  |  |  |  |  | 1 |  |  |  | 1 | 1 |  | 0111  1 | | | | | 1 |  |  | 1 | 1 | 1 | 00 | | | 1 |  |  |  |  |  |  | 1 | 001 | | | | | |  |  | 1 |
|  |  |  |  |  |  |  |  |  |  |  |  |  |  |  |  |  |  |  |  |  |  |  | 0110 | | | | |  |  |  |  |  |  | 01 | | |  |  |  |  |  |  |  |  | 010 | | | | | |  |  |  |
|  |  |  |  |  |  |  |  |  |  |  |  |  |  |  |  |  |  |  |  |  |  |  | 0100 | | | | |  |  |  |  |  |  |  | | |  |  |  |  |  |  |  |  | 000 | | | | | |  |  |  |
|  |  |  |  |  |  |  |  |  |  |  |  |  |  |  |  |  |  |  |  |  |  |  | 0101 | | | | |  |  |  |  |  |  |  | | |  |  |  |  |  |  |  |  | 011 | | | | | |  |  |  |
|  |  |  |  |  |  |  |  |  |  |  |  |  |  |  |  |  |  |  |  |  |  |  | 00?? | | | | |  |  |  |  |  |  |  | | |  |  |  |  |  |  |  |  |  | | | | | |  |  |  |

Symbols: –, indels; β, GATA; ▲, ACTTTTTCTTTTTCCTATTGGAC; δ, GTTAATAATCATAACAACTATCTAT; #, TTTGCT; ■, TTTTTCT; ○, AATCTAAAAATTACTTTTTCTTTTT; ☆, TGTAT ATAAC; *, ATACACAAATAATACACAATAT; ◆, AATTGTATGATCAATGTCTGCTTCTCTTCTATATACATCTTTGTATATAACTGTATATAAC; α, GGGTCGTATTTGTAACCCACTAGGTACAGT

TTTTGACTAAACTGTCATAAAAAAAACTGTGCC; ★, ATACACAAATAATACACAATATTGAATTGTATGATCAATGTCTGCTTCTCTTCTATATACATCTTTGTATATAAC; ω, TCTTGTAAGAGT

AAAAACAAAACAATAAGGTAAGGGACGGAAGAAGTGAAAAGAAACTATCTCTTATTAAGTTTTCAGGAATAGGAAAATATGATGATCGGAACTTGCATTAGATTCTTATTA;
