# Supplementary material for: Phylogeography and allopatric divergence of cypress species (Cupressus L.) in the Qinghai-Tibetan Plateau and adjacent regions
Source: BMC Evol Biol. 2010 Jun 22;10:194. doi: 10.1186/1471-2148-10-194 (PMC3020627; doi:10.1186/1471-2148-10-194)
Supplement: Additional File 4 — Estimates of mutation rate for Cupressus and dates of divergence between the Asian species/lineage estimated by BEAST. [file 1471-2148-10-194-S4.DOC]

**Additional File 4** The estimates of the mutation rate of *Cupressus* and the divergence timescales between the Asian species/lineage

We downloaded from GenBank and aligned three plastid DNA fragments (*trn*D-*trn*T, *trn*S-*trn*G and *trn*L-*trn*F) of three *Cupressus* species, three *Juniperus* species, three *Calitropsis* species, one species from *Platycladus*, *Calocedrus*, *Chamaecyparis* and *Thuja*. We used *Thuja* as outgroup in the BRAST analyses. We constrained the stem lineage of *Chamaecyparis* to be 99.63 millions years ago based on the fossil records of *Cupressinocladus interruptus* (similar to *Chamaecyparis*) in the late Cretaceous to Tertiary (Stockey RA, Kvaček J, Hill RS, Rothwell GW, Kvaček Z. 2005. Fossil record of Cupressaceae s.lat. In: Farjon A, eds. *A monograph of Cupressaceae and Sciadopitys*. Kew, UK: Royal Botanic Gardens, Kew, 54-68). We constrained the stem lineage of *Juniperus* Sect. *Sabina* (represented here by *J. formosana* and *J. drupacea*) to be 38.34 millions years ago based on the fossil record of *J. pauli* (similar to those species of Sect. *Sabina*) in the Eocene/Oligocene boundary (Kvaček Z. 2002. A new juniper from the Palaeogene of Central Europe. *Feddes Repertorium* 113: 492-502). The mutation rate of *Cupressus* was calculated to be 3.2 × 10^-10^ substitutions per site per year.


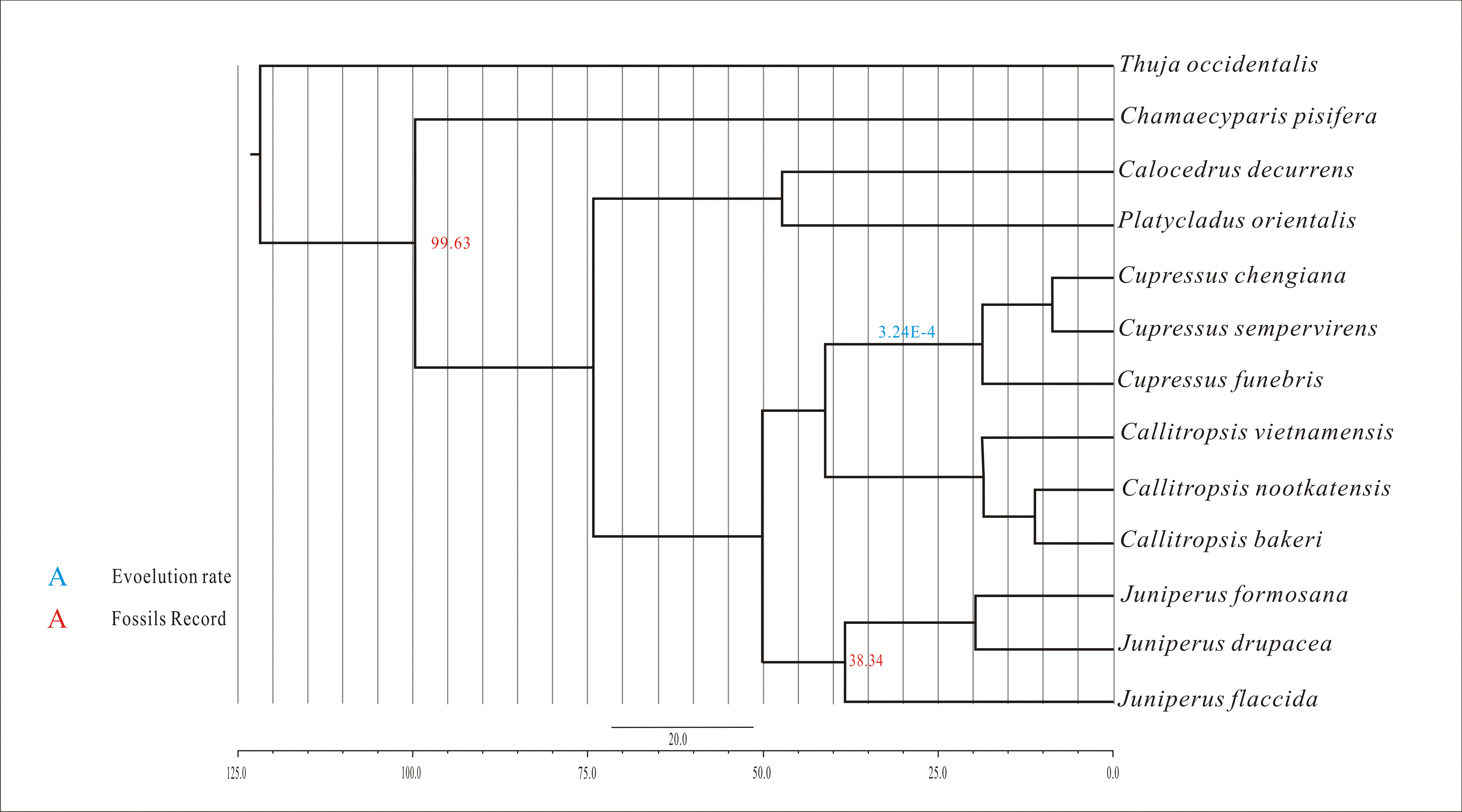


Additional File 4-figure 1: A preliminary estimation of the possible mutation rate (in blue) of *Cupressus* and fossil records (in read with millions years ago).

We then used the mutation rate to estimate the divergences of the Asian cypress species. The timescales of major nodes were showed as follows.


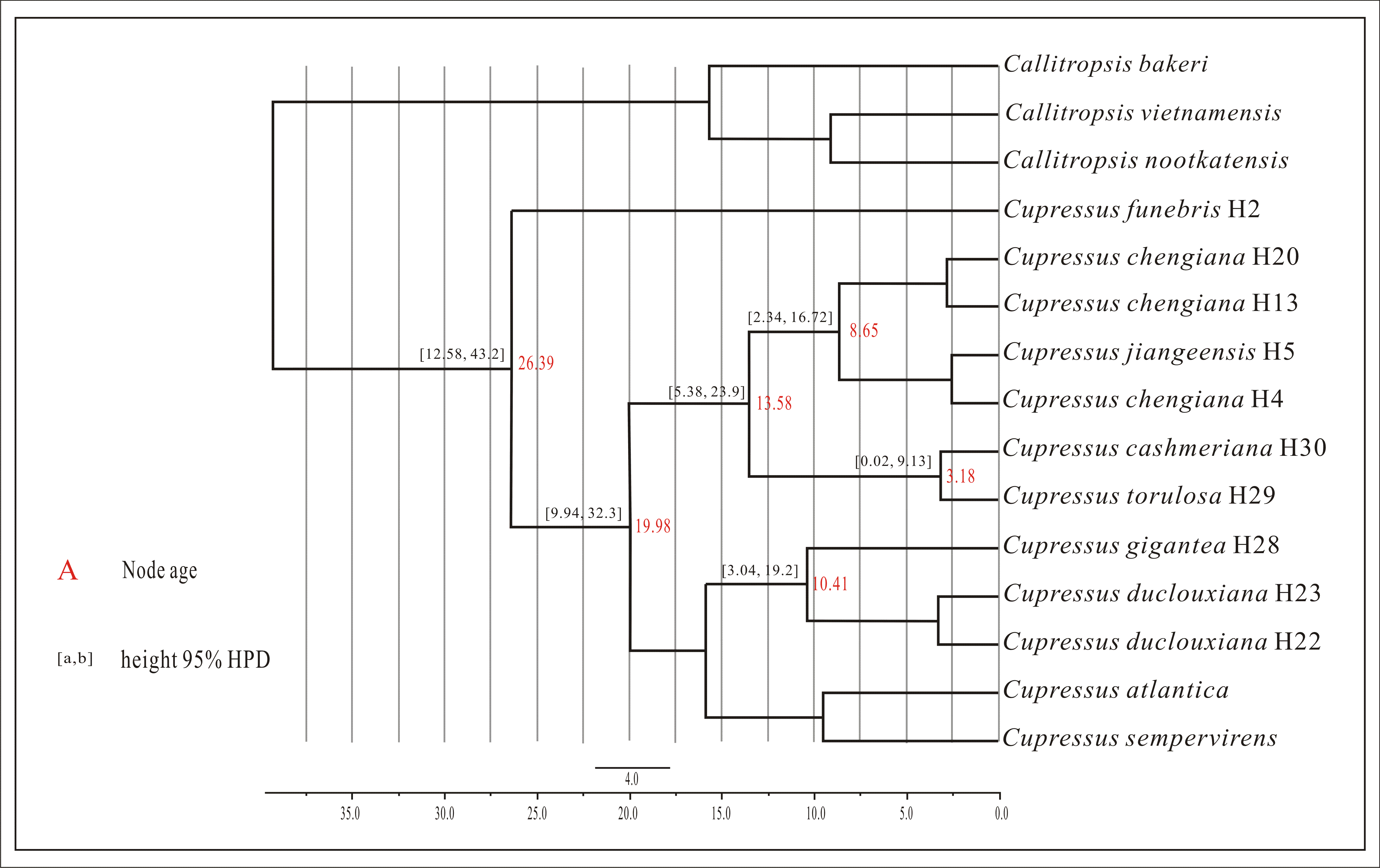


Additional File 4-figure 2: The divergence timescales of the Asian cypress lineage/species (in read with millions years ago).
